# Iron and risk of dementia: Mendelian randomisation analysis in UK Biobank

**DOI:** 10.1136/jmg-2023-109295

**Published:** 2024-01-08

**Authors:** Francesco Casanova, Qu Tian, Janice L Atkins, Andrew R Wood, Daniel Williamson, Yong Qian, David Zweibaum, Jun Ding, David Melzer, Luigi Ferrucci, Luke C Pilling

**Affiliations:** 1 Department of Clinical and Biomedical Sciences, University of Exeter, Exeter, UK; 2 Translational Gerontology Branch Longitudinal Studies Section, National Institute on Aging, Bethesda, Maryland, USA; 3 Department of Medical Imaging, University of Exeter, Exeter, UK

**Keywords:** Dementia, Genetics, Population

## Abstract

**Background:**

Brain iron deposition is common in dementia, but whether serum iron is a causal risk factor is unknown. We aimed to determine whether genetic predisposition to higher serum iron status biomarkers increased risk of dementia and atrophy of grey matter.

**Methods:**

We analysed UK Biobank participants clustered into European (N=451284), African (N=7477) and South Asian (N=9570) groups by genetic similarity to the 1000 genomes project. Using Mendelian randomisation methods, we estimated the association between genetically predicted serum iron (transferrin saturation [TSAT] and ferritin), grey matter volume and genetic liability to clinically defined dementia (including Alzheimer’s disease [AD], non-AD dementia, and vascular dementia) from hospital and primary care records. We also performed time-to-event (competing risks) analysis of the TSAT polygenic score on risk of clinically defined non-AD dementia.

**Results:**

In Europeans, higher genetically predicted TSAT increased genetic liability to dementia (Odds Ratio [OR]: 1.15, 95% Confidence Intervals [CI] 1.04 to 1.26, p=0.0051), non-AD dementia (OR: 1.27, 95% CI 1.12 to 1.45, p=0.00018) and vascular dementia (OR: 1.37, 95% CI 1.12 to 1.69, p=0.0023), but not AD (OR: 1.00, 95% CI 0.86 to 1.15, p=0.97). Higher TSAT was also associated with increased risk of non-AD dementia in participants of African, but not South Asian groups. In survival analysis using a TSAT polygenic score, the effect was independent of apolipoprotein-E ε4 genotype (with adjustment subdistribution Hazard Ratio: 1.74, 95% CI 1.33 to 2.28, p=0.00006). Genetically predicted TSAT was associated with lower grey matter volume in caudate, putamen and thalamus, and not in other areas of interest.

**Discussion:**

Genetic evidence supports a causal relationship between higher TSAT and risk of clinically defined non-AD and vascular dementia, in European and African groups. This association appears to be independent of apolipoprotein-E ε4.

**WHAT IS ALREADY KNOWN ON THIS TOPIC**

- Brain iron deposition is common in dementia, but the role of systemic plasma iron is unclear.

**WHAT THIS STUDY ADDS**

- In this Mendelian randomisation analysis of 468 331 UK Biobank participants, genetic predisposition to higher transferrin saturation has significantly genetic liability to clinically define non-Alzheimer’s dementia but not Alzheimer’s disease, in both European and African groups

**HOW THIS STUDY MIGHT AFFECT RESEARCH, PRACTICE OR POLICY**

- Our results support a causal link between higher transferrin saturation and risk of clinically defined non-Alzheimer’s dementia.

## Background

In the central nervous system, iron availability is important for many biological functions, including neurotransmitter synthesis and myelin production. Excess iron in the brain may have deleterious effects on brain function directly or through interaction with excessive reactive oxygen species resulting in mitochondrial dysfunction and neuroinflammation.^1^ Brain iron accumulation increases with age and the accumulation varies by anatomical location,^2^ and iron accumulation in insoluble amyloid plaques and neurofibrillar tangles is seen in patients with Alzheimer’s disease (AD).^3^ While iron deposition in the brain is the potential pathological mechanism for dementia, general practitioners can only monitor plasma iron, so it is important to investigate the causal role of this modifiable risk factor.

In the iron-overload disease hereditary haemochromatosis (which affects males more severely than females), males homozygous for the p.C282Y variant in gene ‘homeostatic iron regulator’ (*HFE*) have increased risk of dementia^4^ but no increased risk of AD specifically,^5^ compared with non-carriers. In the general population, genetic variants associated with plasma iron are not associated with the risk of AD,^6^ yet whether genetic variants associated with higher iron have a causal relationship with non-AD dementias in the general population is unknown.

Mendelian randomisation (MR) methods can assess the causal effect of an exposure on disease risk^7^ using genetic variants identified from genome-wide association studies (GWAS) in the general population. A recent GWAS of 250 000 people identified genetic variants associated with serum iron status biomarkers^8^; this included transferrin saturation (TSAT) and ferritin, the main plasma iron markers used clinically.^9^ Transferrin binds and transports iron in the blood, and TSAT is the estimated % of transferrin with bound iron, whereas ferritin is crucial for intracellular iron storage. Plasma ferritin and TSAT fluctuate over time, influenced by factors such as dietary intake and infections, while genetic variants are fixed at conception and convey lifetime exposure to higher/lower levels. MR exploits the random distribution of alleles at birth to minimise the risk of reverse causation and the effect of unmeasured confounders; MR can estimate causal associations using genetic variants as instruments (proxies) for the exposure.^10^

In this study, we aimed to examine the association between genetically predicted serum iron levels (TSAT and ferritin) and risks of clinically defined all-cause dementia, AD and non-AD in the UK Biobank (UKB) study. In secondary analysis, we investigated vascular dementia specifically as well as grey matter volumes, tested whether the association of TSAT with dementia is independent of apolipoprotein E ε4 (*APOE-4*) carrier status, and determined whether the associations were also observed in different genetic ancestry groups.

## Methods

### Population

UKB recruited over 500 000 individuals aged 40–70 years at baseline in 2006–2010, from England, Scotland and Wales, and collected detailed information from all participants, via questionnaires, interviews, physiological measurements and genetic data.^11^

We analysed data from 451 284 participants genetically similar to the 1000 genome project European Ancestry superpopulation (‘EUR-like’), with additional analysis of participants genetically similar to African Ancestry superpopulation (AFR-like) and South Asian Ancestry superpopulation (SAS-like) (similarity defined by using principal component analysis (PCA); see online supplemental methods). From 2014 onwards, a subgroup of 100 000 participants is undergoing an additional assessment visit that includes an MRI scan,^12^ with 39 748 available at the time of analysis.

10.1136/jmg-2023-109295.supp1 Supplementary data

### Genetic variants associated with TSAT and ferritin

For TSAT, we used 20 out of the 21 genome-wide significant (p<5×10^−8^) independent variants from the latest GWAS^8^ (UKB did not contribute any data to this GWAS because TSAT was not measured in UKB); one variant (rs7165401) was excluded due to significant deviation from Hardy-Weinberg Equilibrium (HWE) (online supplemental table 1). For serum ferritin, we used 65 of the 69 genome-wide significant variants from the same GWAS, with 4 removed due to minor allele frequency (MAF) <0.1% in UKB (online supplemental table 1). Median-estimated F statistics were 44.7 for TSAT and 41.6 for ferritin (online supplemental table 1). These were calculated from betas and SEs using established methods.^13^

10.1136/jmg-2023-109295.supp2 Supplementary data

### Ascertainment of dementia diagnosis in UKB

Dementia diagnoses were ascertained from Hospital Episode Statistics, available in the whole cohort up to September 2021 (diagnoses recorded as International Classification of Disease [ICD] version 9 and ICD-10 codes). Primary care (general practice, GP) data were available in 45% of the cohort (recorded as Read2 or CTV3 codes), up to 2016 or 2017 depending on data provider (https://biobank.ndph.ox.ac.uk/ukb/label.cgi?id=3000).

Our primary outcomes were ‘all-cause dementia’, AD and non-AD dementia (ie, ‘all-cause dementia’ excluding participants who received an AD diagnostic code). For AD and non-AD dementia, the reference group are participants with no dementia diagnosis. We also performed secondary exploratory analysis of vascular dementia. More details of the phenotypes can be found in online supplemental methods and online supplemental table 2.

### Grey matter volumes in UKB

We examined grey matter volumes as secondary outcomes in a subset of participants who had available brain MRI data after the study entry (median difference between MRI assessment and baseline 9.2 years). The UKB brain MRI data have been processed centrally and imaging-derived phenotypes made available to analysts (https://open.win.ox.ac.uk/ukbiobank/big40/).^14^ In this study, we used data on grey matter volumes using a larger sample size than previously published brain imaging phenotypes.^15^ We examined grey matter volumes in multiple regions of interest, including subcortical (amygdala, caudate, hippocampus, pallidum, putamen and thalamus), frontal (inferior frontal gyrus, middle frontal gyrus, superior frontal gyrus, precentral gyrus and supplementary motor cortex), parietal (superior parietal lobe, precuneus, postcentral gyrus) and temporal (inferior temporal gyrus, middle temporal gyrus and parahippocampal gyrus) areas as previously described.^16^ Grey matter volumes in specific regions of interest were the sum of right and left hemisphere. We adjusted for brain volume as a covariate. More details of grey matter volume derivation are found in online supplemental material.

### GWAS in UKB

Genetic associations with dementia were tested using ‘Scalable and Accurate Implementation of Generalized mixed model 1’ V.0.35, which controls for unbalanced case–control ratios and for sample relatedness using mixed-model approaches. Genetic associations for quantitative traits (ie, inverse-normalised MRI phenotypes) were tested using ‘BOLT-LMM’ 2 V.2.3.2, which performs linear mixed-effects models to efficiently control for sample relatedness. UKB-imputed genotypes (V.3 data release, n=93 million) were analysed. GWAS were adjusted for age, sex, study centre (1–22) and genotyping microarray (2) at run time. Variants with imputation quality (INFO) <0.3, MAF <0.1% or significant deviation from HWE (p<5×10-8) were excluded, leaving ~16 million for subsequent analysis. Summary statistics for these GWAS are available to download from FigShare (DOI 10.6084/m9.figshare.21828498). Patients with missing data were excluded from analysis. A summary of the phenotypes, sample sizes, case numbers and the number of genetic variants passing inclusion criteria is available in online supplemental table 3.

### MR analysis

In this study, we used two different MR approaches, to exploit the advantages that they convey. We first used summary statistics MR (also known as ‘two-sample MR’) to estimate the main causal effect and perform sensitivity analyses such as exploring whether pleiotropy affects our results and, second, we used a polygenic score to perform stratified analyses (described in the online supplemental methods).

We used the inverse-variance weighted (IVW) method to estimate the causal effect of the genetically predicted exposures on the genetically predicted outcomes.^17^ More details on IVW are found in online supplemental methods.

All analyses were performed in R (V.4.2.1) using the ‘MendelianRandomization’ R package (V.0.6.0).

### Cumulative risk analysis

To compliment the MR IVW approach, we used the TSAT polygenic score in UKB participants (see online supplemental methods for details of derivation) to estimate the cumulative risk (incidence) of dementia diagnosis by age. We used competing risk survival analysis to adjust for the competing risk of mortality (if unaccounted for, this can result in overestimation of the cumulative risk). We used R (V.4.2.1) with packages ‘survival’ (V.3.4-0) and ‘tidycmprsk’ (V.0.2.0) to perform analyses and ‘ggplot2’ (V.3.3.6) for plotting.

### Survival analysis

We used the TSAT polygenic score in UKB participants to estimate the association with dementia using competing risk (Fine-Gray) time-to-event analysis, accounting for the competing risk of mortality. We used R (V.4.2.1) package ‘tidycmprsk’ (V.0.2.0). An advantage of this ‘one-sample MR’ approach is that covariates such as *APOE ε4* status (defined by rs429358 and rs7412) can be included. The model was also adjusted for age at assessment, sex, assessment centre, genotyping microarray (Axion or BiLEVE) and genetic principal components of ancestry 1–10. Unless otherwise specified, the analysis was performed in the EUR-like group (n=451 284).

### Association between TSAT polygenic score and dementia in AFR-like and SAS-like groups

We repeated the MR analysis testing the association between genetically predicted TSAT and dementia (all-cause, AD and non-AD) in the two most common groups by genetic ancestry in UKB after those in the EUR-like group: the AFR-like and SAS-like groups. Due to the low number of cases in these ancestry groups, we limited our analysis to using the polygenic score for TSAT as exposure (rather than an underpowered individual SNP-based analysis using IVW). All ancestry groups were defined using PCA (see online supplemental methods for details).

### MR sensitivity analysis

#### Leave-one-out analysis

For traits where a significant association was found, we performed a leave-one-out analysis using the ‘TwoSampleMR’ R package to test if any of the SNPs used in our analysis had a disproportionately large effect on outcomes. Leave-one-out analysis removes one SNP at the time from the exposure SNP list and repeats the analysis in each subset iteratively; SNP with a large effect will affect the overall estimate when removed.

#### Egger/weighted median

We performed a sensitivity analysis with pleiotropy-resistant MR methods MR-Egger and weighted median estimator.^18^

### Data availability

UKB data are available to any bona fide researcher following application (https://www.ukbiobank.ac.uk/enable-your-research/apply-for-access).

Summary statistics for these GWAS are available to download from FigShare^19^ (DOI 10.6084/m9.figshare.21828498).

## Results

In the analysis of UKB participants in the EUR-like group (n=451 284), 7016 participants were diagnosed with dementia in the available medical records up to September 2021. Summary statistics for the participants including AD and non-AD dementias are presented in table 1. In the AFR-like group (total N=7958), there were 125 dementia cases and in the SAS-like group (total N=10 198), there were 131.

**Table 1:** Summary statistics for UK Biobank cohorts under analysis

		Ancestral group*		
		EUR-like	AFR-like	SAS-like
Participants included	n (%)	451 284	7958	10 198
Sex, female	n (%)	244 887 (54.3)	4612 (58.0)	4801 (47.1)
Age at baseline	Min:max	40:70	40:70	40:70
	Mean (SD)	56.8 (8.0)	51.9 (8.1)	53.5 (8.5)
Age end follow-up†	Min:max	41:85	43:84	43:85
	Mean (SD)	69.6 (8.0)	64.4 (8.0)	66.1 (8.4)
Dementia diagnosis				
All-cause	n (%)	7016 (1.6)	125 (1.6)	131 (1.3)
Alzheimer’s disease	n (%)	3040 (0.7)	53 (0.7)	48 (0.5)
Non-Alzheimer’s disease‡	n (%)	3976 (0.9)	72 (0.9)	83 (0.8)
Vascular	n (%)	1538 (0.3)	29 (0.4)	28 (0.3)

All dementia cases diagnosed after baseline.

*Grouping defined by genetic principal component analysis (see the Methods section).

†Age of participant at hospital inpatient record censoring date (for most participants, 30 September 2021) or age at death, if participant died before this.

‡Received a non-Alzheimer’s dementia diagnosis and did not ever receive an Alzheimer’s disease-related diagnosis.

AFR-like, African Ancestry superpopulation; EUR-like, European Ancestry superpopulation; SAS-like, South Asian Ancestry superpopulation.

### Associations between genetically predicted serum TSAT and ferritin with clinically defined AD and non-AD dementias in EUR-like participants

**Figure 1:**
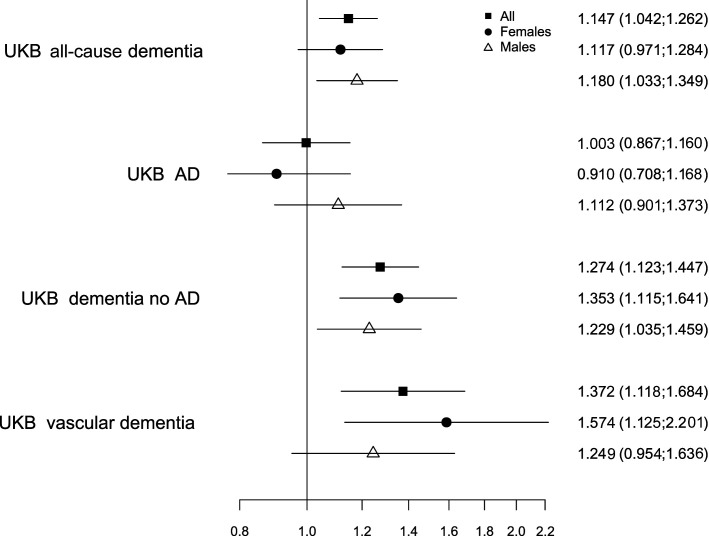
Genetically predicted transferrin saturation (TSAT) associations with dementia diagnosis in the UK Biobank (UKB) EUR-like group. Results are from Mendelian randomisation (inverse-variance weighted) analysis. Twenty TSAT genetic variants are from Moksnes *et al,* 8 with outcome associations from analysis in the UKB EUR-like group. Estimates are expressed as the OR per SD in genetically predicted TSAT (95% CIs). See online supplemental table 5 and 9 for detailed results, including sensitivity analyses. AD, Alzheimer’s disease; EUR-like, European Ancestry superpopulation.

Using MR, we estimated that 1 SD higher genetically predicted serum TSAT was associated with higher genetic liability to clinical diagnosis of all-cause dementia (OR per SD of genetically predicted TSAT 1.15; 95% CI 1.04 to 1.26, p=0.0051; figure 1 and online supplemental table 4). One SD increase in genetically predicted TSAT was associated with higher genetic liability of non-AD dementia (OR 1.27; 95% CI 1.12 to 1.45, p=1.8×10^−4^), but not with AD (OR 1.00; 95% CI 0.87 to 1.16, p=0.968). The latter result was consistent when using independent AD data from a large published GWAS (OR 1.00; 95% CI 0.98 to 1.01, p=0.934).^20^ Multiple statistical testing was not considered because the sex-stratified and dementia subtype analyses were not independent of the primary analysis.

In secondary analysis, genetically predicted TSAT was associated with genetic liability to vascular dementia (figure 1 and online supplemental table 4). Associations between genetically predicted TSAT, genetic liability to non-AD dementia and vascular dementia appeared to be stronger in women than men (figure 1 and online supplemental table 4), but formal comparison using Fisher’s Z showed no significant sex differences (p for difference>0.05).

There was no association between genetically predicted ferritin and genetic liability to dementia in UKB (online supplemental table 5).

### Time-to-event analysis of TSAT polygenic score on non-AD dementia clinical diagnosis accounting for competing risk of mortality and *APOE* ε4 genotype

**Figure 2:**
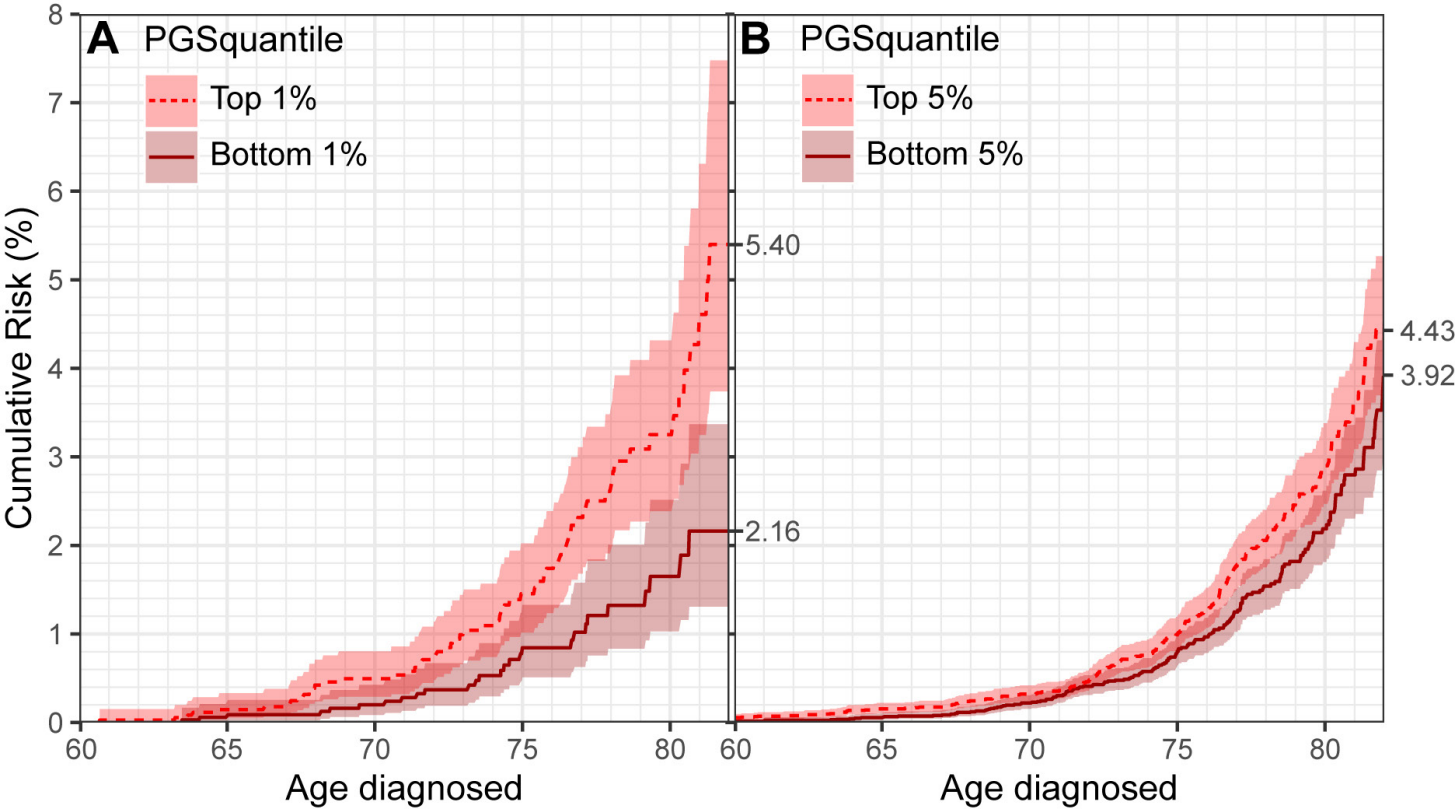
Cumulative risk of non-Alzheimer’s dementia, stratified by quantile of transferrin saturation polygenic score. Panel A shows the top 1% of polygenic score compared to the bottom 1% and Panel B shows the top 5% of polygenic score compared to the bottom 5%. Analysis in UK Biobank participants, adjusted for the competing risk of mortality. Shaded areas are the 95% CIs. X-axis restricted to 82 years, as very few participants attained age >82 years during follow-up. PGS, polygenic score for transferrin saturation.

In addition to the analyses described above using summary statistics-based MR methods (IVW approach), we also generated a polygenic score for TSAT in the UKB participants for additional individual-level analyses. First, we estimated the cumulative risk of clinically defined non-AD dementia in quantiles of TSAT polygenic score, adjusted for the competing risk of mortality. Participants in the top 1% of TSAT polygenic score had 5.4% (95% CI 3.7% to 7.5%) risk of non-AD dementia risk by age 82 years (too few participants attain ages >82 in the follow-up to give reliable estimates) compared with 2.2% (95% CI 1.3% to 3.4%) risk of non-AD dementia by age 82 years in the participants in the bottom 1% of TSAT polygenic score (figure 2A), that is, 3.2% excess. We also repeated the analysis comparing cumulative risk using the larger quantiles: top 5% of TSAT polygenic score versus the bottom 5% (figure 2B), and for deciles and quintiles (online supplemental figure 1).

In time-to-event analysis (competing risks regression) of the TSAT polygenic score on risk of clinically defined non-AD dementia, the association was independent of *APOE* ε4 (without adjustment subdistribution HR (sHR) 1.59; 95% CI 1.23 to 2.05, p=0.0003; with adjustment for *APOE* ε4 sHR 1.74; 95% CI 1.33 to 2.28, p=0.00006, all sHRs per SD in TSAT polygenic score).

### Association between TSAT polygenic score and clinically defined dementia in participants in the AFR-like and SAS-like groups

The mean age of the participants in the AFR-like and SAS-like groups was significantly lower than the EUR-like participants (p<0.05), yet proportions of clinically diagnosed with dementia were similar across the groups despite the lower attained ages at the end of follow-up (table 1). Correspondingly, the cumulative risk of dementia by age 82 years was greatest in the AFR-like group and lowest in the EUR-like group (see online supplemental figure 2).

We used the inverse normalised polygenic score for TSAT (within each genetic ancestry group, due to different distributions between populations (see online supplemental figure 3)) in time-to-event analysis (competing risks regression) separately in different groups. For the analysis of all-cause dementia in the AFR-like group, the effect was larger than in the EUR-like group (for AFR-like sHR 1.31 SD in TSAT polygenic risk score, 95% CI 1.09 to 1.57, p=0.0037; for EUR-like sHR 1.03, 95% CI 1.01 to 1.06, p=0.012, table 2), while no effect was observed in the SAS-like group (p>0.05). Similar patterns were observed for non-AD dementia diagnoses, while there was no association using AD dementia as outcome in all ancestry groups (table 2).

**Table 2:** Association between transferrin saturation (TSAT) polygenic score and dementia diagnosis in different genetic ancestry groups in UK Biobank

Outcome	Genetic ancestry group	sHR (95% CI)	P value
	EUR-like	1.03 (1.01; 1.06)	0.012
All-cause dementia	AFR-like	1.31 (1.09; 1.57)	0.0037
	SAS-like	1.03 (0.86; 1.24)	0.74
	EUR-like	1.05 (1.02; 1.07)	0.0008
Non-Alzheimer’s dementia	AFR-like	1.29 (1.05; 1.58)	0.016
	SAS-like	1.03 (0.85; 1.26)	0.76
	EUR-like	1.00 (0.965; 1.04)	0.91
Alzheimer’s disease	AFR-like	1.13 (0.86; 1.49)	0.38
	SAS-like	1.23 (0.91; 1.67)	0.18

sHR per SD of the inverse normalised TSAT polygenic score from competing risks regression time-to-event analysis accounting for the competing risk of mortality, adjusted for age at assessment, sex, assessment centre and genetic principal components.

AFR-like, African Ancestry superpopulation; EUR-like, European Ancestry superpopulation; SAS-like, South Asian Ancestry superpopulation; sHR, subdistribution HR.

There was no association between ferritin polygenic score and all-cause dementia in the AFR-like (sHR 1.00, 95% CI 0.84 to 1.20, p=0.96) or SAS-like (sHR 1.04, 95% CI 0.87 to 1.25, p=0.65) groups (we used the inverse normalised polygenic score for ferritin within each genetic ancestry group).

### Associations between genetically predicted serum TSAT and ferritin with grey matter volumes

**Figure 3:**
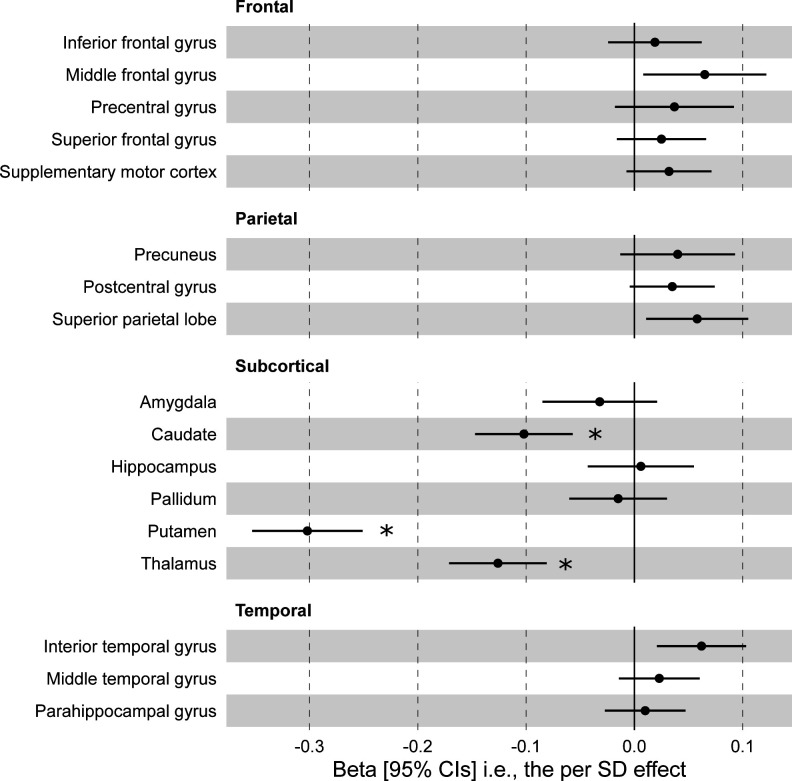
Genetically predicted transferrin saturation (TSAT) associations with grey matter volumes in the UK Biobank EUR-like group. Results are from Mendelian randomisation (inverse-variance weighted) analysis. Twenty TSAT genetic variants are from Moksnes *et al,* 8 with outcome associations from analysis in the UK Biobank EUR-like group. Estimates are expressed as unit change per SD in genetically predicted TSAT (95% CI). See online supplemental table 6 for detailed results, including sensitivity analyses. EUR-like, European Ancestry superpopulation. * = statistically significant after multiple testing correction (17 tests. *p* < 0.003).

Using MR, we found that higher genetically predicted TSAT in EUR-like participants was associated with lower genetically predicted grey matter volumes in subcortical regions including caudate, putamen and thalamus (figure 3 and online supplemental table 6). These associations remained significant after correction for multiple statistical testing (Bonferroni-adjusted p value threshold for seven regions <0.007). We did not find significant association with other regions after multiple testing correction (online supplemental table 6). These analyses were not performed in other ancestry groups due to small sample sizes with MRI data. There was an association between genetically predicted ferritin and genetically predicted grey matter volumes in putamen (online supplemental table 7).

### Sensitivity analysis

Details are available in the online supplemental tables 8 and 9. In brief, removing rs79220007 in the *HFE* gene (a proxy for rs1800562, that is, *HFE* p.C282Y) from MR IVW estimates affected the significance but not effect size for some results, and we found no evidence for pleiotropy (MR-Egger intercept p>0.05).

## Discussion

In this longitudinal analysis of clinically defined dementia diagnosis in UKB participants, we found that higher genetically estimated TSAT (that is, greater lifetime exposure) is associated with increased genetic liability to non-AD dementia in the EUR-like group, supporting a causal link. We show that higher genetically predicted TSAT is associated with decreased genetically predicted grey matter volume in specific regions. Our analysis showed no effect of TSAT on AD risk, using data from UKB and separately using summary statistics from a recent large published GWAS of AD.^20^ We also found that TSAT polygenic score was associated with dementia diagnosis in UKB participants in the AFR-like group.

A recent systematic review found limited evidence linking iron intake and dementia.^21^ Yet, we recently reported that the iron overload disease hereditary haemochromatosis is associated with an increased risk of dementia in male homozygotes carriers of the p.C282Y mutation in the *HFE* gene.^4^ We extend our previous findings and show that genetically determined TSAT in the general population is also associated with dementia genetic liability. Our results support a causal association between iron and dementia in a community cohort. The TSAT genetic instruments were derived from a GWAS of the general population,^8^ and are consistent when variants in the *HFE* gene are excluded. These results appear to conflict with those from the Nutrition Examination Survey I Epidemiologic Follow-up Study, where measured TSAT was not associated with increased odds of dementia,^22^ but our data represent exposure to higher plasma TSAT over a lifetime, as opposed to one-off measurement used in epidemiological longitudinal models.

Our AD results are in agreement with previous work reporting null associations^6^ despite evidence that iron-related neuroinflammation is involved in neurodegenerative process.^1^ We find that genetically predicted TSAT increased genetic liability to non-AD dementia (and vascular dementia), which suggests that peripheral iron might affect blood vessels and might not be as important in AD. This is supported by evidence of endothelial dysfunction in iron overload^23^ and the association between cortical iron and white matter microstructure.^24^ We could not fully test this hypothesis in UKB because white matter hyperintensity, an MRI phenotype considered to be a marker of small-vessel disease, is only available for the whole brain, with no regional data available to us.

The effect of TSAT polygenic score on dementia is different between groups, with significant effects on participants in the AFR-like and EUR-like, but not the SAS-like group. Though these results should be interpreted with caution (we used a polygenic score derived in Europeans (as defined by authors), and therefore its applicability in different ancestry groups requires further study),^25^ this suggests the association seen in the EUR-like group may extend to other ancestry groups. Because we did not find any non-EUR-like generated TSAT polygenic score, we reported the MAF in the different groups in UKB (online supplemental table 1), which can be considered in the interpretation of our results. While using a polygenic score derived in EUR-like groups has clear limitations, Mukadam *et al* showed that a dementia polygenic score derived from EUR-like populations can be used to quantify genetic risk in people from more diverse ancestry groups.^26^

Our data on the secondary outcomes of brain volumes show that genetically higher TSAT results in smaller genetically predicted grey matter volume in the basal ganglia, important for motor function. Previous studies have shown brain atrophy in these areas is associated with increased iron content in the same area.^27^ Future studies should further examine the association between plasma iron and genetically predicted TSAT with iron content in the brain. Grey matter atrophy is a hallmark of cognitive impairment, and our data support a role of plasma iron in this pathway leading to dementia. No other grey matter areas of interest were found to be associated with TSAT after multiple test correction. These neuroimaging findings provide mechanism insights on the effect of genetically predicted TSAT on dementia, particularly non-AD dementia and vascular dementia.

Our results found no effect of genetically higher plasma ferritin on genetic liability to dementia outcomes analysed, and it appears that the effect of ferritin on grey matter volume is less marked than the effect of TSAT, with reduction in volume observed only in putamen. Ferritin has been found to be involved in AD pathology and neuroinflammatory processes,^28 29^ but these results tend to derive from cerebral fluid ferritin while our data are limited to plasma ferritin. Taken together, our plasma TSAT and ferritin results suggest that the iron storage capacity in blood (ferritin) might be less important in the development of dementia than the amount of iron bound (TSAT), not excluding a role for iron-related molecules in other tissues and fluids.

Our study has limitations that need to be acknowledged. First, the UKB is a volunteer cohort and is healthier than the general population.^30^ Second, our results rely on hospital and GP records to ascertain dementia cases. It is possible that cases can be misclassified given the multifactorial nature of dementia and the high prevalence of mixed dementia,^31^ so our AD and non-AD groups might not be entirely accurate; future studies are needed to validate our findings. Additionally, GP data were available for only 45% of the cohort. Detailed analysis of other dementia subtypes (eg, dementia with Lewy bodies) was not possible due to limitations in the diagnostic code resolution (see the Methods section). Third, while a biological explanation might support our data, caution should be exercised in interpreting our results due to the common occurrence of mixed features in dementia, and the possibility that unrecognised and unclassified subtype of dementia can confound our results. Finally, we acknowledge the limitation of applying a polygenic score derived in one group to other principal component-defined ancestry groups. In this study, we aimed to apply the principles outlined in the recent report from the National Academies of Sciences, Engineering, and Medicine^32^ to the best of our current possibilities, but future studies should move away from discrete ancestry clusters to a continuum of genetic ancestries.^33^

We have shown that genetically determined higher TSAT is associated with increased genetic liability to non-Alzheimer’s dementia, supporting a causal association. Our results suggest that lifetime exposure to higher TSAT is associated with grey matter atrophy in caudate, putamen and thalamus subcortical regions.

## Data Availability

Results are available in a public, open access repository. Access to UK Biobank participant level data requires application. UKB data are available to any bone fide researcher following application (https://www.ukbiobank.ac.uk/enable-your-research/apply-for-access). Summary statistics for these GWAS are available to download from FigShare (DOI 10.6084/m9.figshare.21828498).
