# Supplementary material for: Iron and risk of dementia: Mendelian randomisation analysis in UK Biobank
Source: J Med Genet. 2024 Jan 8;61(5):435–42. doi: 10.1136/jmg-2023-109295 (PMC11041574; doi:10.1136/jmg-2023-109295)
Supplement: Supplementary data [file jmg-2023-109295supp001.pdf]

Serum iron and risk of dementia: Mendelian randomization analysis in UK Biobank

Dr Francesco Casanova, *et al*

Supplementary Information:

Supplementary Methods 2
Ascertainment of dementia diagnosis in UK Biobank 2
Derivation of grey matter volume phenotypes 2
Mendelian randomization (MR) IVW estimate and standard error 2
Polygenic score derivation 3
Defining Groups in UK Biobank using Principal Component Analysis 3
Supplementary Results 4
Sensitivity analysis 4
Leave one out analysis 4
MR Egger and Median 4
Supplementary Figures 5
SFig 1: Cumulative risk of non-Alzheimer's dementia, stratified by quantile of transferrin saturation polygenic score 5
SFig 2: Cumulative risk of all-cause dementia, stratified by ancestry group 6
SFig 3: Distribution of transferrin saturation polygenic score, stratified by ancestry group . 7
SFig 4: Scatter plot of individual transferrin saturation-associated genetic variant effects on all-cause dementia diagnosis in EUR-like group 8
SFig 5: Scatter plot of individual transferrin saturation-associated genetic variant effects on non-Alzheimer dementia diagnosis in EUR-like males. 9
SFig 6: Scatter plot of individual transferrin saturation-associated genetic variant effects on vascular dementia diagnosis in EUR-like group (all participants). 10
SFig 7: Scatter plot of individual transferrin saturation-associated genetic variant effects on vascular dementia diagnosis in EUR-like group (female participants). 11
SFig 8: Scatter plot of individual transferrin saturation-associated genetic variant effects on gray matter volume in the caudate in EUR-like males 12
Supplementary Tables 13
References 14

Casanova F, *et al. J Med Genet* 2024; 61:435–442. doi: 10.1136/jmg-2023-109295

BMJ Publishing Group Limited (BMJ) disclaims all liability and responsibility arising from any reliance placed on this supplemental material which has been supplied by the author(s)

Supplementary Methods

Ascertainment of dementia diagnosis in UK Biobank

Specific diagnostic codes for dementia were identified from the UK NHS National Institute for Health and Care Excellence (NICE) Quality and Outcomes Framework (QOF) Business Rules (https://qof.digital.nhs.uk), version 37.0 (version date 09/06/2017). Read v2 and CTV3 codes were converted to ICD-9 and ICD-10 codes using UK Biobank Resource 592 (Clinical coding classification systems and maps). A detailed list of ICD-9, ICD-10, Read2 and CTV3 codes used can be found in Supplementary Table 2.

We also performed secondary exploratory analysis of vascular dementia. We were unable to analyze “dementia with Lewy bodies” or frontotemporal dementia due to very low number of cases ascertained. UK Biobank only provided the first 5 digits of the diagnostic codes, rendering us unable to differentiate some subtypes (for example, ICD-10 code G31.83 “Dementia with Lewy bodies” is 6 digits but the HES diagnosis data only includes G31.8 i.e., the first 5 digits). Patient with missing data were excluded from analysis.

Derivation of grey matter volume phenotypes

Grey matter volume regions were created as the sum of the left and right hemisphere using data from UK Biobank, except when multiple data fields were aggregated into one phenotype (details below).. For subcortical regions the amygdala was created as the sum of data fields 25888 and 25889, caudate 25880 and 25881, hippocampus 25886 and 25887, pallidum 25884 and 25885, putamen 25882 and 25883 and thalamus 25878 and 25879. For frontal regions the inferior frontal gyrus was created as the sum of data fields 25790 to 25793, middle frontal gyrus 25788 and 25789, precentral gyrus 25794 and 25795, superior frontal gyrus 25786 and 25787and supplementary motor cortex 25832 and 25833. For parietal regions precuneus was created as the sum of data fields 25842 and 25843, postcentral gyrus 25814 and 25815, superior parietal lobe 25816 and 25817. For temporal regions interior temporal gyrus was created as the sum of data fields 25808 to 25813, middle temporal gyrus 25802 to 25807 and parahippocampal gyrus 25848 to 25851. All resulting phenotypes were adjusted for brain volume (data field 25009)

Mendelian randomization (MR) IVW estimate and standard error

MR relies on several assumptions:

the exposure SNPs are robustly associated with the relevant measured exposure. This is quantified by the F-statistic, which can be approximated by the ratio of the SNP-exposure association estimate, $\hat{\beta}$ and its standard error, SE($\hat{\beta}$), squared (equation 1)[3].

$$F = \left(\frac{\hat{\beta}}{SE(\hat{\beta})}\right)^2$$

the exposure SNPs are not associated with confounding factors that bias conventional epidemiological associations.

the exposure SNPs are only associated with the outcome through the risk factor.

For IVW the causal estimate β is:

$$\beta_{(ivw)} = \frac{\sum_{n=1}^{N} X_n Y_n \sigma_{Yn}^{-2}}{\sum_{n=1}^{N} X_n^2 \sigma_{Yn}^{-2}}$$

and the approximate standard error (se) of the estimate is:

$$se_{(\beta ivw)} = \sqrt{\frac{1}{\sum_{n=1}^{N} X_n^2 \sigma_{Yn}^{-2}}}$$

where Xn is the estimate of the genetic association with the risk factor with standard error σXn and Yn is the estimate of the genetic association with the outcome with standard error σYn, for each of the N genetic variants (n=1,…,N).

Polygenic score derivation

We derived a polygenic score for transferrin saturation in the UKB participants by summing the number of TSAT-increasing alleles carried, multiplied by the known effect of the allele on TSAT levels [4]. In mathematical notation the polygenic score $\hat{S}$ is derived:

$$\hat{S} = \sum_{n=1}^{N} X_n \hat{\beta}_n$$

where X_n is the number of TSAT-increasing alleles weighted by their published effect $\hat{B}_n$, for each of the N genetic variants $(n = 1 \dots N)$. Patient with missing data were excluded from analysis.

Defining Groups in UK Biobank using Principal Component Analysis

To determine genetic groups of individuals in the UK Biobank, we performed principal components (PC) analysis using samples from the 1000 Genomes Project (1GP) [5]. Individuals from the UK Biobank were subsequently projected into the 1GP PC space using the SNP loadings derived from the initial PC analysis to minimise confounding of PC values due to varying degrees of relatedness within UK Biobank. Next, we calculated the means of the first 4 PCs stratified by individuals known to be from each of the 5 main genetic ancestral superpopulations in the 1GP data: European (EUR), South Asian (SAS), East Asian (EAS), African (AFR) and Admixed American (AMR). Using the means derived from the 1GP reference dataset, we subsequently performed K-means clustering analyses to determine which individuals from UK Biobank could be classified as EUR-like, SAS-like or AFR-like. Using 1GP ancestry specific mean values for PCs 1-4, we classified 451,454 individuals in UK Biobank to be similar to EUR genetic ancestry. Next, using the 1GP mean values described above for principal components 2-4, we classified 10,207 individuals to be similar to SAS genetic ancestry. Finally, using 1GP means for PCs 1-4, we identified 7,968 individuals estimated to be similar to AFR genetic ancestry. In the manuscript we refer to these groups defined by similarity to 1GP as EUR-like, SAS-like and AFR-like groups.

Casanova F, *et al. J Med Genet* 2024; 61:435–442. doi: 10.1136/jmg-2023-109295

Supplementary Results

Sensitivity analysis

Leave one out analysis

Results of the leave one out analysis (Supplementary table 8) using all-cause dementia as outcome revealed that rs79220007 in the *HFE* gene (a proxy for rs1800562 i.e., *HFE* p.C282Y) had a strong effect on the analysis in all participants where the effect was reduced and became not significant (OR without this SNP 1.14: 95%CI 0.99-1.32, p= 0.068, Supplementary Figure 4). Similarly, when using non-AD dementia as our outcome rs79220007 reduced the effect in males (OR 1,16 95%CI 0.90-1.50, p= 0.24 Supplementary Figure 5). rs79220007 in the *HFE* gene also had a strong effect on our exploratory secondary analysis in vascular dementia, with no significant effect observed when removing the SNP in all participant and females only analysis (Supplementary Figure 6 and 7).

MR Egger and Median

Results using pleiotropy resistant methods were generally consistent with the IVW results (Supplementary table 9). For the TSAT to dementia phenotypes analysis the point estimate for the Egger method was directionally consistent and of the same magnitude, but the CIs crossed the null (not uncommon, as the Egger method has reduced power compared to IVW due to variable intercept). Weighted median analysis confirmed the association between genetically predicted TSAT and non-AD dementia. There was no evidence that pleiotropy (as assessed by Egger intercept p-value) was affecting our results.

Casanova F, *et al. J Med Genet* 2024; 61:435–442. doi: 10.1136/jmg-2023-109295

Supplementary Figures

SFig 1: Cumulative risk of non-Alzheimer's dementia, stratified by quantile of transferrin saturation polygenic score

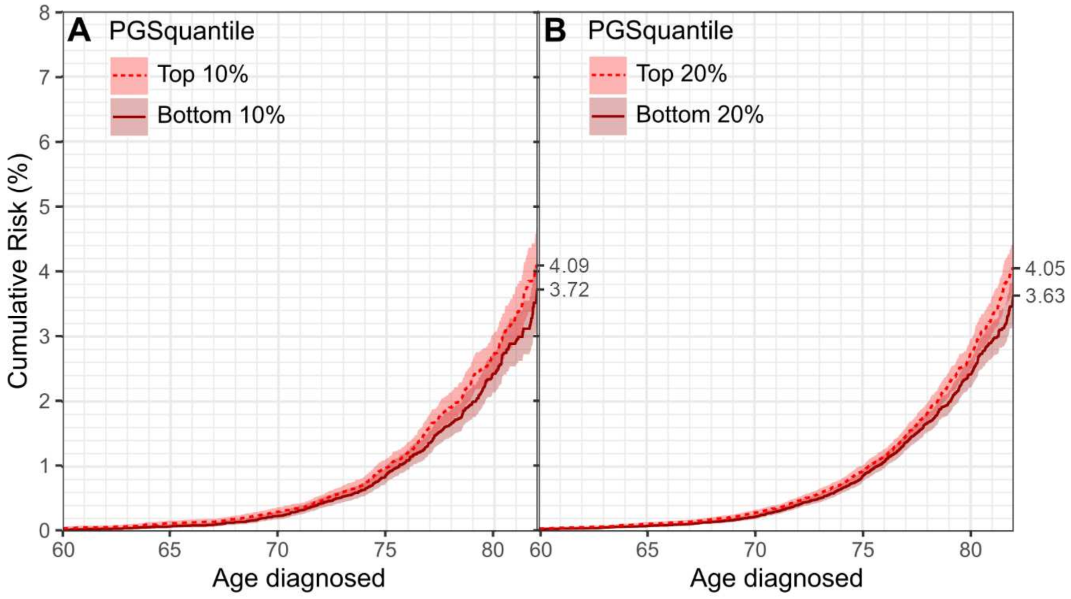

Analysis in UK Biobank participants, adjusted for the competing risk of mortality. X-axis restricted to 82 years, as very few participants attained age >82 during follow-up. PGS = polygenic score for transferrin saturation.

SFig 2: Cumulative risk of all-cause dementia, stratified by ancestry group

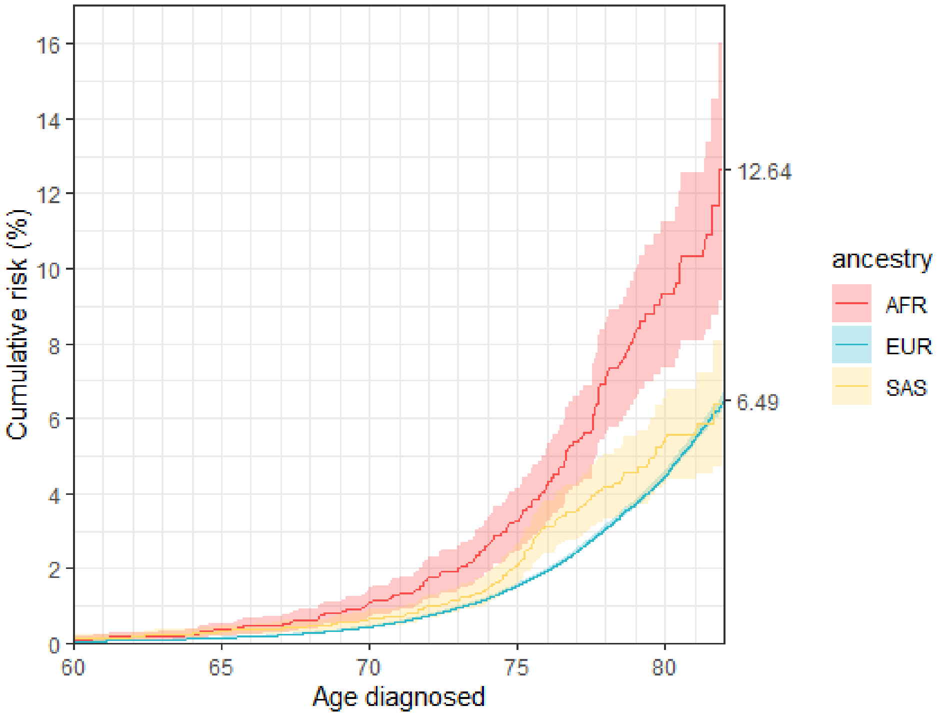

Analysis in UK Biobank participants, adjusted for the competing risk of mortality. X-axis restricted to 82 years, as very few participants attained age >82 during follow-up. AFR=African-like group, EUR=European-like, SAS=South Asian-like. Other ancestry groups were too small to study.

SFig 3: Distribution of transferrin saturation polygenic score, stratified by ancestry group

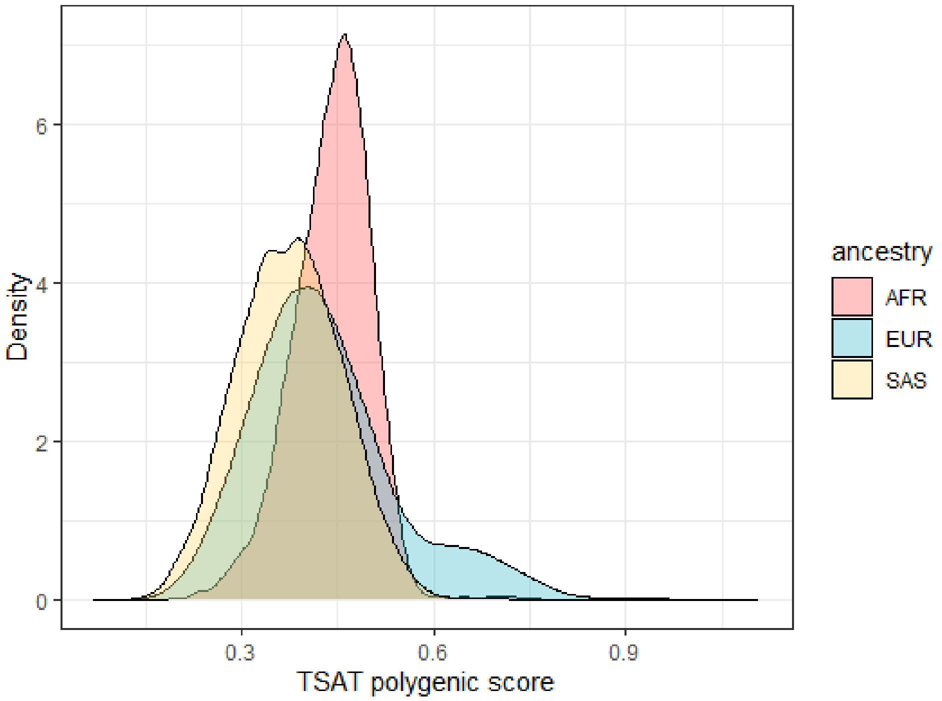

Despite (expected) differences in allele frequency between ancestry groups there is substantial variation between individuals. AFR=African-like, EUR=European-like, SAS=South Asian-like. Other ancestry groups were too small to study.

Casanova F, *et al. J Med Genet* 2024; 61:435–442. doi: 10.1136/jmg-2023-109295

SFig 4: Scatter plot of individual transferrin saturation-associated genetic variant effects on all-cause dementia diagnosis in EUR-like group

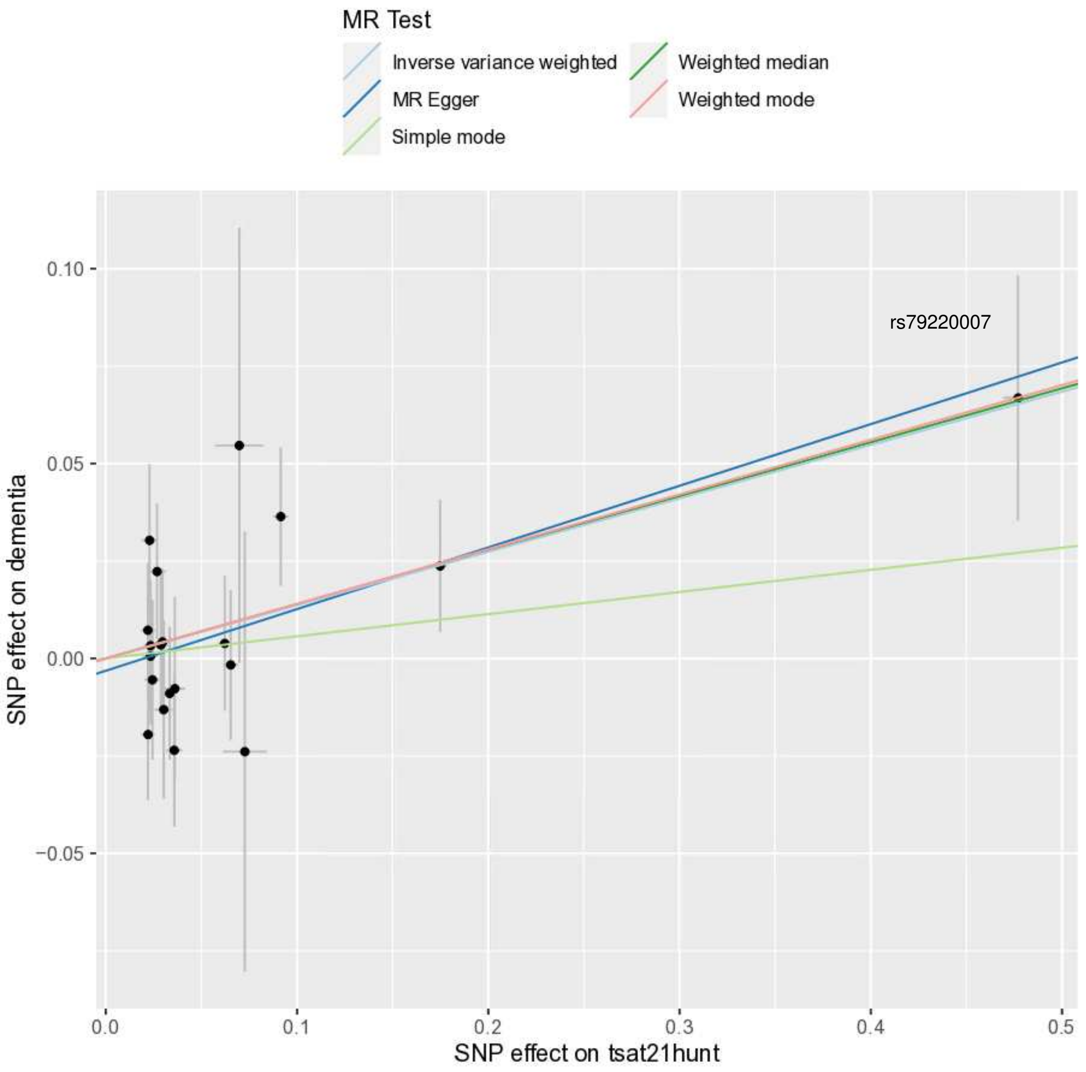

SFig 5: Scatter plot of individual transferrin saturation-associated genetic variant effects on non-Alzheimer dementia diagnosis in EUR-like males.

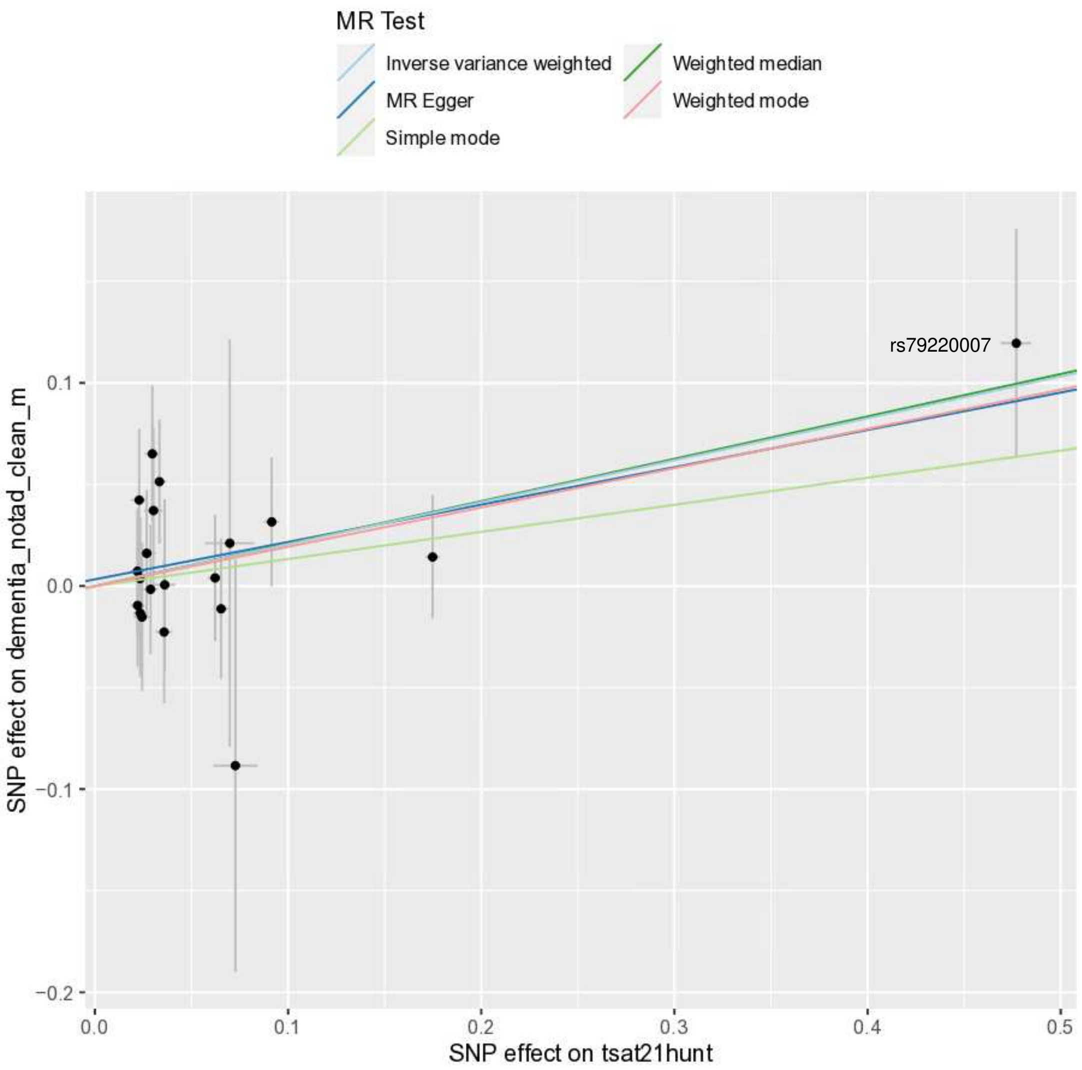

Casanova F, *et al. J Med Genet* 2024; 61:435–442. doi: 10.1136/jmg-2023-109295

SFig 6: Scatter plot of individual transferrin saturation-associated genetic variant effects on vascular dementia diagnosis in EUR-like group (all participants).

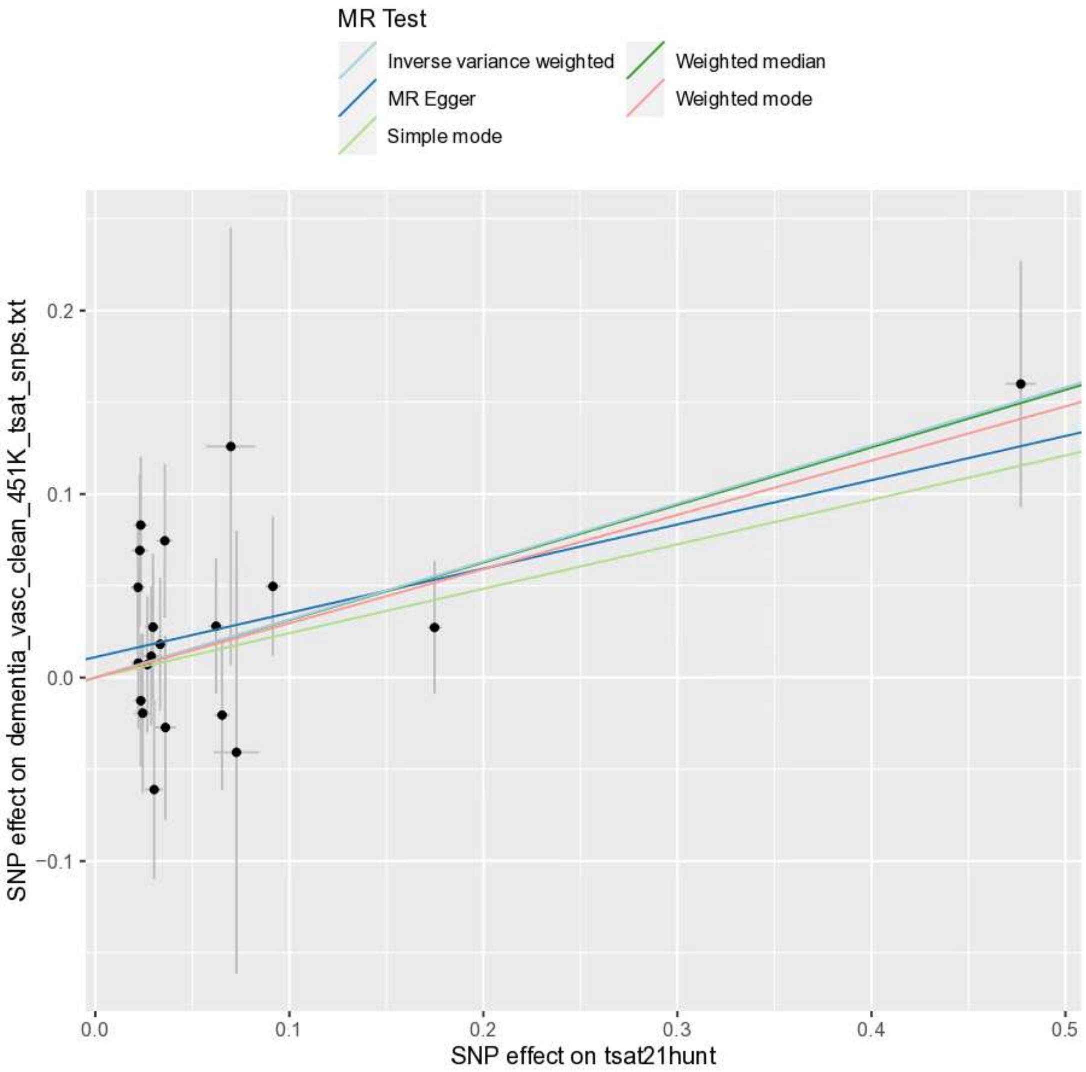

Casanova F, *et al. J Med Genet* 2024; 61:435–442. doi: 10.1136/jmg-2023-109295

SFig 7: Scatter plot of individual transferrin saturation-associated genetic variant effects on vascular dementia diagnosis in EUR-like group (female participants).

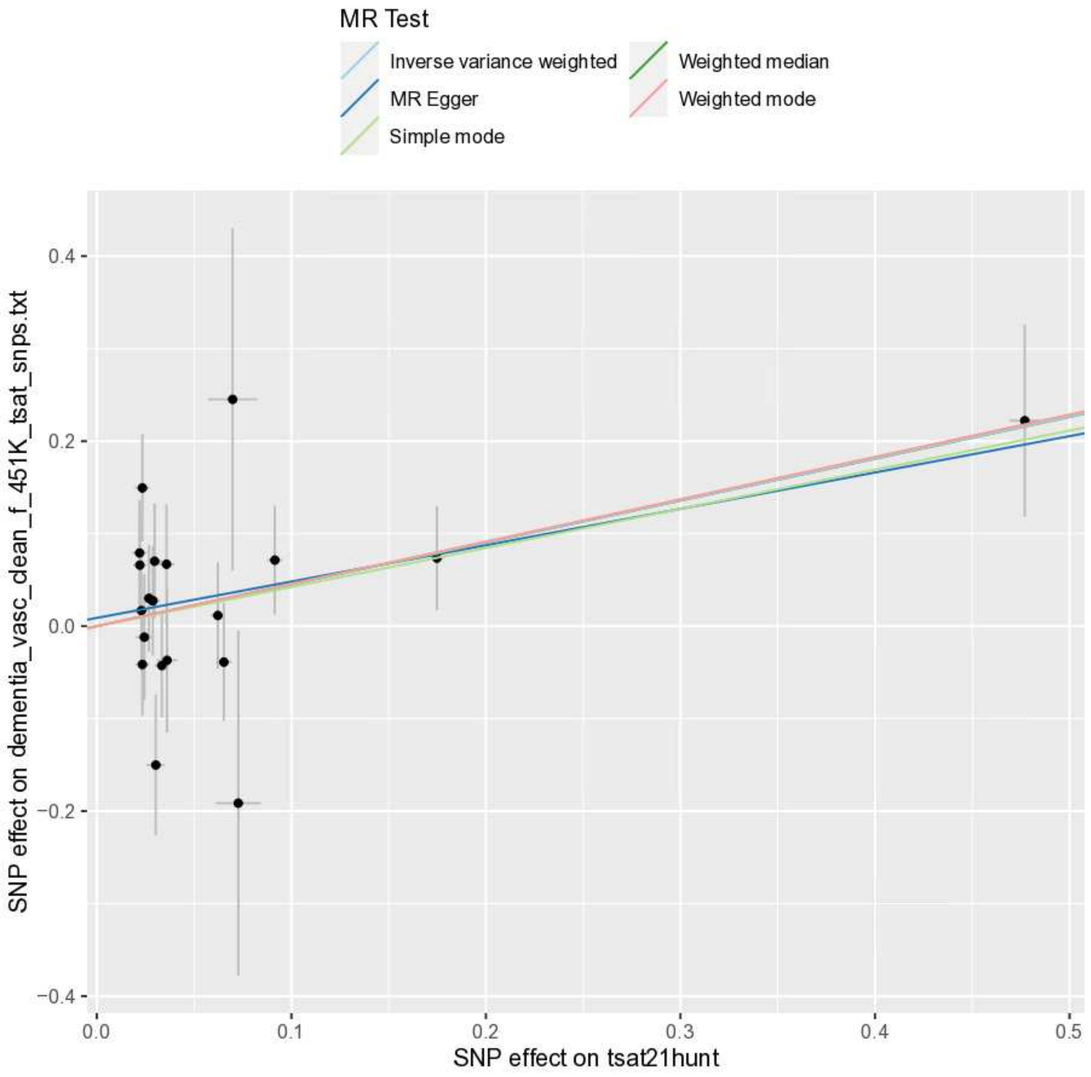

Casanova F, *et al. J Med Genet* 2024; 61:435–442. doi: 10.1136/jmg-2023-109295

SFig 8: Scatter plot of individual transferrin saturation-associated genetic variant effects on gray matter volume in the caudate in EUR-like males.

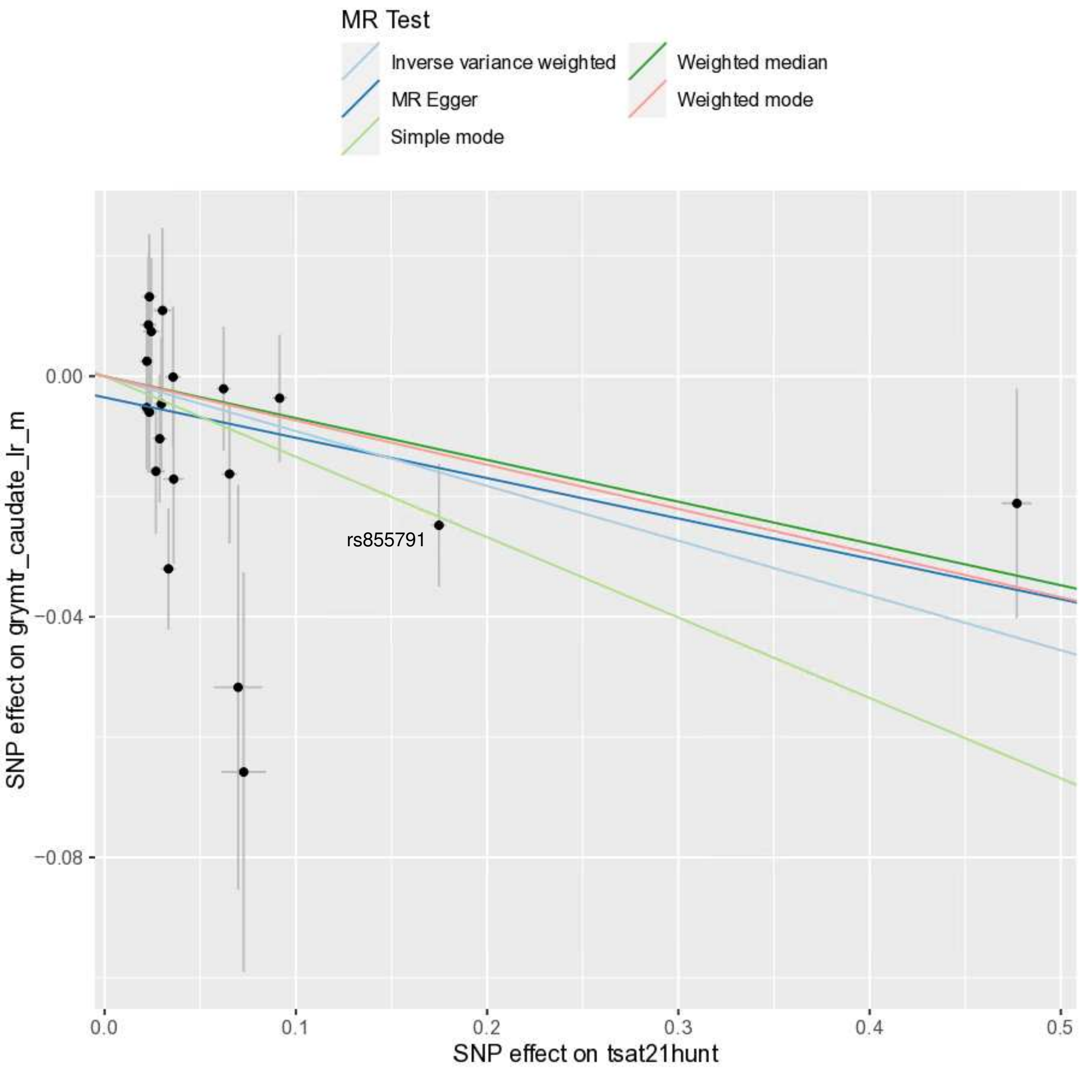

Casanova F, *et al. J Med Genet* 2024; 61:435–442. doi: 10.1136/jmg-2023-109295

Supplementary Tables

Many Supplementary Tables are too large to appear in this document. We therefore include the Supplementary Tables as a separate Excel document.

Genome-wide association study (GWAS) summary statistics are available to download from FigShare (DOI 10.6084/m9.figshare.21828498). Additional meta information in Supplementary Table 3.

References

1. Zhou W, Nielsen JB, Fritsche LG, et al. Efficiently controlling for case-control imbalance and sample relatedness in large-scale genetic association studies. *Nat Genet* 2018; **50**(9): 1335-41.
2. Loh PR, Tucker G, Bulik-Sullivan BK, et al. Efficient Bayesian mixed-model analysis increases association power in large cohorts. *Nat Genet* 2015; **47**(3): 284-90.
3. Bowden J, Del Greco MF, Minelli C, et al. Improving the accuracy of two-sample summary-data Mendelian randomization: moving beyond the NOME assumption. *Int J Epidemiol* 2019; **48**(3): 728-42.
4. Moksnes MR, Graham SE, Wu KH, et al. Genome-wide meta-analysis of iron status biomarkers and the effect of iron on all-cause mortality in HUNT. *Commun Biol* 2022; **5**(1): 591.
5. Fairley S, Lowy-Gallego E, Perry E, Flicek P. The International Genome Sample Resource (IGSR) collection of open human genomic variation resources. *Nucleic Acids Res* 2020; **48**(D1): D941-D7.

Casanova F, *et al. J Med Genet* 2024; 61:435–442. doi: 10.1136/jmg-2023-109295
